# An inexpensive programmable optogenetic platform for controlled neuronal activation regimens in *C. elegans*

**DOI:** 10.1063/1.5120002

**Published:** 2020-01-02

**Authors:** Zachary Crawford, Adriana San-Miguel

**Affiliations:** Department of Chemical and Biomolecular Engineering, North Carolina State University, Raleigh, North Carolina 27695, USA

## Abstract

In *Caenorhabditis elegans*, optogenetic stimulation has been widely used to assess neuronal function, control animal movement, or assay circuit responses to controlled stimuli. Most studies are performed on single animals and require high-end components such as lasers and shutters. We present an accessible platform that enables controlled optogenetic stimulation of *C. elegans* in two modes: single animal stimulation with locomotion tracking and entire population stimulation for neuronal exercise regimens. The system consists of accessible electronic components: a high-power light-emitting diode, Arduino board, and relay are integrated with MATLAB to enable programmable optogenetic stimulation regimens. This system provides flexibility in optogenetic stimulation in freely moving animals while providing quantitative information of optogenetic-driven locomotion responses. We show the applicability of this platform in single animals by stimulation of cholinergic motor neurons in *C. elegans* and quantitative assessment of contractile responses. In addition, we tested synaptic plasticity by coupling the entire-population stimulation mode with measurements of synaptic strength using an aldicarb assay, where clear changes in synaptic strength were observed after regimens of neuronal exercise. This platform is composed of inexpensive components, while providing the illumination strength of high-end systems, which require expensive lasers, shutters, or automated stages. This platform requires no moving parts but provides flexibility in stimulation regimens.

## I. INTRODUCTION

Human life expectancy has significantly increased worldwide in the past century, and this trend is expected to continue (Lassonde *et al.*, 2017). More people are living longer, and thus age-related diseases, such as neurodegenerative disease, are becoming a growing issue. Neurodegenerative diseases reduce cognitive function by impairing synaptic function and plasticity (Arancio and Chao, 2007; Gispen and Biessels, 2000; Shankar *et al.*, 2008). However, we are far from understanding how neurodegenerative disease drives synapse loss and malfunction. Synaptic plasticity forms the basis of current models for memory, learning, and sensory adaptation (Fox and Stryker, 2017; Hebb, 1949). Behavioral training in Sprague Dawley rats and regulated neuronal activation in *Tritonia diomedea, Xenopus* tadpoles, and *Caenorhabditis elegans* (Abbott and Nelson, 2000; Foster and Dumas, 2001; Hoerndli *et al.*, 2015; Katz *et al.*, 1994) has been shown to improve synaptic plasticity. Studying neuronal exercise is vital for the understanding of the underlying mechanisms of synaptic plasticity, discovering features of learning and memory, and identifying the genes and pathways that play a role in age-associated synaptic plasticity decline.

The nematode *C. elegans* is a prime model organism for manipulating and studying neural circuitry (Sengupta and Samuel, 2009). Adult *C. elegans* have 302 neurons, which provides much simpler neural circuits than vertebrate and mammalian models, particularly the human brain that contains almost 100 billion neurons. The *C. elegans* connectome has been fully mapped to the synaptic level (White *et al.*, 1986). The stereotypical connectivity exhibited by this model system enables informed studies concerning neurogenesis, the functioning of neuronal networks, and neurodegeneration, to name some (Hobert, 2013, 2005; Margeta *et al.*, 2008; Piggott *et al.*, 2011). *C. elegans'* self-fertilization makes them conducive to the generation of large isogenic populations, thereby enabling simple culturing for high-throughput studies. Most importantly, *C. elegans* is optically transparent, which allows for *in vivo* imaging of cellular and subcellular structures and neuronal stimulation using optogenetics. Optogenetics is a technique allowing for control of neurons using light-sensitive proteins (Deisseroth, 2011). The use of light-gated ion channels in neurons allows neuronal activation and behavioral control of an organism by light stimulation. A wide array of behaviors can be achieved depending on where the light-sensitive protein is expressed. The transparency of *C. elegans* enables *in vivo* optogenetic stimulation, and this has been applied in several ways (Fischer *et al.*, 2018). Most systems have used custom built systems that incorporate costly lasers and shutters (Leifer *et al.*, 2011; Stirman *et al.*, 2011). While optogenetic applications have included the measurement of muscular forces on chip (Qiu *et al.*, 2015), stimulation of touch receptor neurons (Husson *et al.*, 2012; Rabinowitch *et al.*, 2016a), activation at presynaptic (Weissenberger *et al.*, 2011), on-chip contraction (Stirman *et al.*, 2010), and simultaneous stimulation and tracking (Gengyo-Ando *et al.*, 2017). These systems, however, require costly equipment and most are only capable of single-animal stimulation.

Alternative approaches to develop inexpensive platforms for optogenetic stimulation of *C. elegans* have also been undertaken. These platforms, however, do not enable simultaneous stimulation and imaging and are limited in scope with the amount of data that can be extracted (Kawazoe *et al.*, 2013; Pokala and Glater, 2018; Rabinowitch *et al.*, 2016b). Despite the litany of applications and wide use of optogenetics to test neuronal function in *C. elegans*, the optogenetic toolset has not yet been applied to create a simple, economical system capable of specific neuronal stimulation in large populations while also enabling quantitative characterization of single animal's responses.

In this work, we present a platform for optogenetic-driven neuronal stimulation that includes the use of light-emitting diode (LED) lamps, MATLAB, and an Arduino controller to automate neuronal exercise. Current systems for neuronal stimulation typically involve expensive light sources, such as lasers, high power projectors, and mercury lamps. These light sources are not highly configurable or long-lasting and often require either the use of mechanical shutters or costly pattern generators for controlled short pulses of light exposure (Hoerndli *et al.*, 2015; Weissenberger *et al.*, 2011). Both LEDs and Arduino boards are long-lasting and open to easy customization, enabling our platform to provide a large variety of exposure options. LEDs are now commonly used in microscopy and, as mentioned earlier, have also been used for optogenetics (Rabinowitch *et al.*, 2016b). We incorporate LEDs as a low-cost reliable light source for optogenetic stimulation in a flexible platform that can operate in several modes: single-animal stimulation and contraction analysis, whole population stimulation on plate, and stimulation and analysis in liquid media. We incorporate image processing to quantitatively assess the locomotion responses of optogenetic stimulation in *C. elegans* motoneurons and apply our platform to assess the effects of neuronal stimulation on synaptic function and plasticity. In contrast to other platforms, our system enables higher resolution analysis of locomotion due to higher framerates and higher resolution of images acquired. In addition, our system allows for a wide variety of fast, programmable stimulation regimens due to higher temporal exposure capabilities (exposure resolution is 5 ms). Our platform also allows for simultaneous stimulation and live characterization of single animals' contractile responses in a quantitative manner by incorporating image processing algorithms to analyze videos and extract quantitative information of animals' responses to optogenetic stimulation.

## II. RESULTS

### A. Automated platform for high throughput optogenetic-driven exercise

We constructed a platform that enables optogenetic stimulation and quantitative analysis of behavioral responses in *C. elegans* by integrating inexpensive components (LED, Arduino board) with image processing algorithms. This platform can also stimulate multiple animals simultaneously, thus enabling higher throughput analysis of the effects of optogenetic-driven neuronal stimulation in *C. elegans.* A key component of this platform is the use of LEDs for optogenetic stimulation. Optogenetic-driven neuronal stimulation requires high frequency light exposures, for which LEDs are well-suited. Conventional mercury lamps and lasers have a warm-up time that requires the use of a mechanical shutter or digital micromirror devices, which are minimally customizable and highly expensive (Leifer *et al.*, 2011; Liewald *et al.*, 2008; Stirman *et al.*, 2011). In contrast, LEDs enable immediate illimitation and activation without the need of mechanical components. LEDs have a warmup time on the order of nanoseconds, up to a 50 000-h bulb life, and are cheaper than mercury lamps or laser sources, making them optimal for a customizable and long-term platform (Steuer Costa, 2010). LEDs also have tight wavelength emission ranges, and so neither thermotaxis-inducing infrared radiation nor mutation-inducing ultraviolet radiation should be an issue. The LED lamp used had a dominant emitted wavelength between 450 and 465 nm, with a peak output of ∼457 nm. The royal blue color was chosen since both channelrhodopsin variants used have a peak and maximally steady response at ∼450 nm (Lin, 2010). The triemitter configuration was chosen to help uniformly illuminate the plate which worms were exercised on. A Pocket Laser Power Meter (840011) by Sper Scientific was used to characterize the light intensity distribution achieved. The meter recorded power across a 9 mm diameter sensor, which was used to estimate intensity at different areas of the plate by dividing by the power by the sensor area. The power meter was calibrated to a wavelength of 633 nm, and therefore, the power readings were corrected to the lamp's wavelength of ∼450 nm by multiplying by a correction factor of 3.29 provided by the manufacturer. The power meter is accurate within 5% at the calibration wavelength of 633 nm, and this accuracy was assumed for our lamp's wavelength spectrum (450 nm–465 nm). Measurements were taken with and without the plate being wrapped in aluminum foil at the center and edge of the plate (Fig. S1), as seen in Table I.

**TABLE I.** LED lamp power and intensity under varying plate conditions.

	Power without aluminum foil	Corrected average intensity (without aluminum foil)	Power with aluminum foil	Corrected average intensity (with aluminum foil)
Plate center	105.28 ± 5.26 mW	1.65 ± 0.08 mW/mm^2^	103.64 ± 5.18 mW	1.63 ± 0.08 mW/mm^2^
Plate midpoint	47.94 ± 2.40 mW	0.75 ± 0.038 mW/mm^2^	75.34 ± 3.77 mW	1.18 ± 0.059 mW/mm^2^
Plate edge	1.97 ± 0.10 mW	0.03 ± 0.002 mW/mm^2^	18.75 ± 0.94 mW	0.29 ± 0.15 mW/mm^2^

The presence of aluminum foil greatly increases the light intensity at the edge of the plate, while keeping the intensity approximately constant in the center. The meter used to take these measurements had a unidirectional sensor, which implies the true light intensity at any point on the plate lies above the listed values. There is some variance in intensity across the plate, but the intensity delivered to the majority of the plate falls close to the value required to activate the channelrhodopsins within the worm strains (Lin, 2010). An additional measurement halfway between the center and the edge of the plate resulted in a corrected intensity of 1.18 ± 0.059 mW/mm^2^. Worm locomotion over the course of a stimulation regimen will lead to animals receiving an average intensity between the maximum and minimum values listed in Table I. In reality, animals have a preference to roam on the bacterial lawn, localized to the center of the plate (between the midpoint and center of Table I), where the intensity variation is not drastic.

**FIG. 1.**
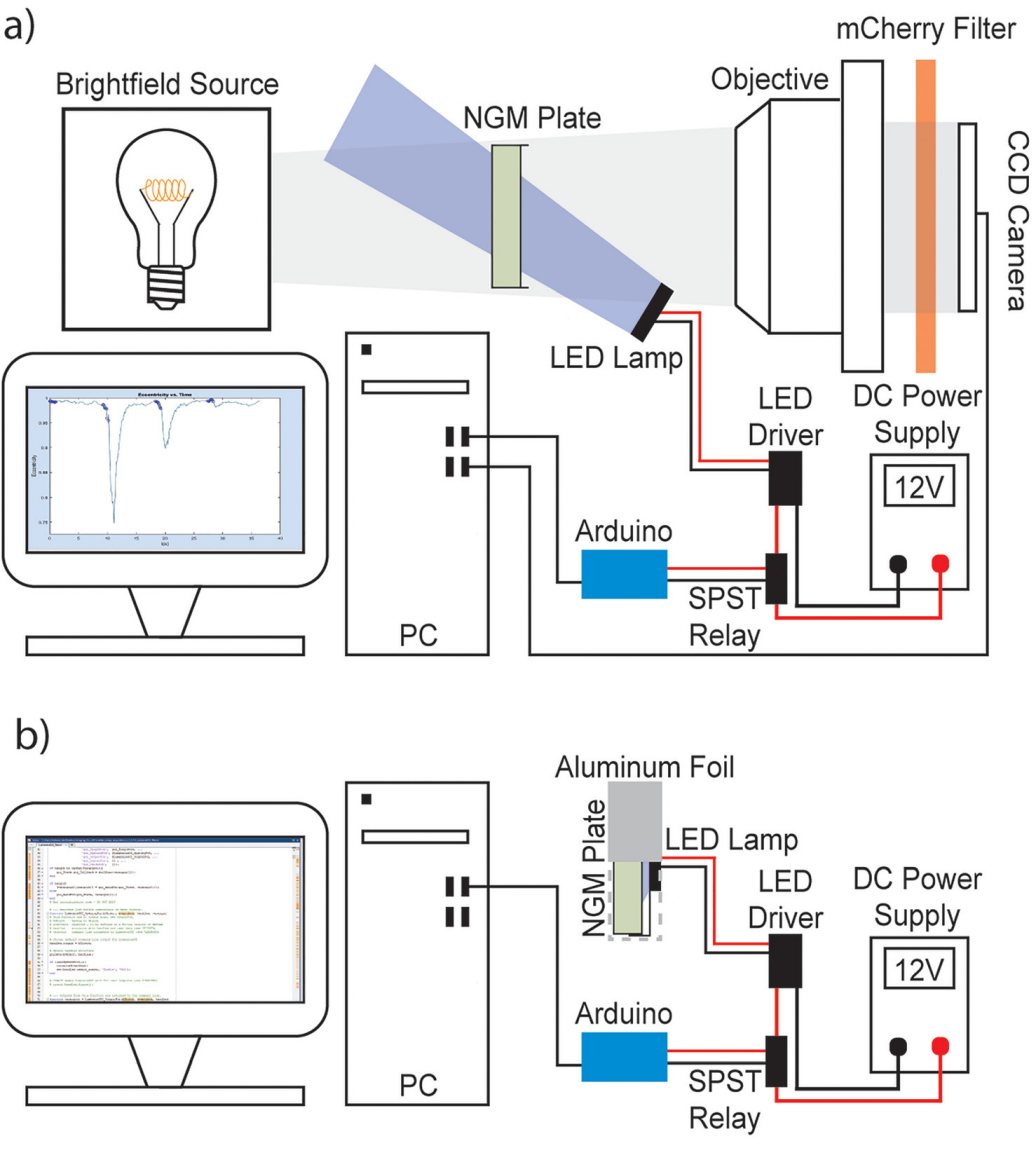
Overview of stimulation and imaging platform. (a) Single-animal stimulation and tracking. Worms are illuminated from below using a brightfield source and imaged at 4× magnification, optionally illuminated with the LED lamp. Imaging procedures include intermittent lamp use, and neuronal stimulation regimens require only the PC, Arduino, DC power supply, and LED lamp. For imaging procedures, a MATLAB program simultaneously sends signals to the Arduino microcontroller and records images via the CCD camera. When the Arduino receives the signal to power the LED lamp, it activates the relay, which routes power from the power supply through the LED driver to the lamp. The LED driver provides the LED lamp with a constant current. The system is placed on a stereoscope, with base brightfield illumination, and an objective and CCD camera at the top. The diagram represents the light path and information flow; microscope components appear rotated 90° counterclockwise in this schematic. (b) Whole population stimulation. The setup is the same as in (a), except no imaging is performed, and the plate is covered with aluminum foil.

The Arduino is connected to the personal computer (PC) for control and data flow purposes via a universal serial bus (USB) cable, but the PC is only capable of supplying 5.0 V DC through the USB port. When the Arduino was used to power the LED lamp, a highly decreased intensity was observed. To ensure the LED lamp maintained a constant power consumption and illuminance, we incorporated 12 V DC power supply. The Arduino was still used to control the LED lamp's on/off status through use of a single-pole single-throw (SPST) relay. The DC power supply was used to provide 12 V DC to the SPST relay, which output to a 350 mA constant-current regulated LED driver, providing a consistent 1.65 W LED lamp power output, as shown in Figs. 1 and S1. When worms were exercised with this system, the lamp was placed face-down on the plate lid, and the entire plate and light were wrapped with aluminum foil to decrease intensity loss. When exposing the worms to light and imaging, an mCherry emission filter with a maximum transmittance at 610 nm assisted with downstream image processing by maintaining a constant luminous intensity.

### B. Liquid media locomotion profile

**FIG. 2.**
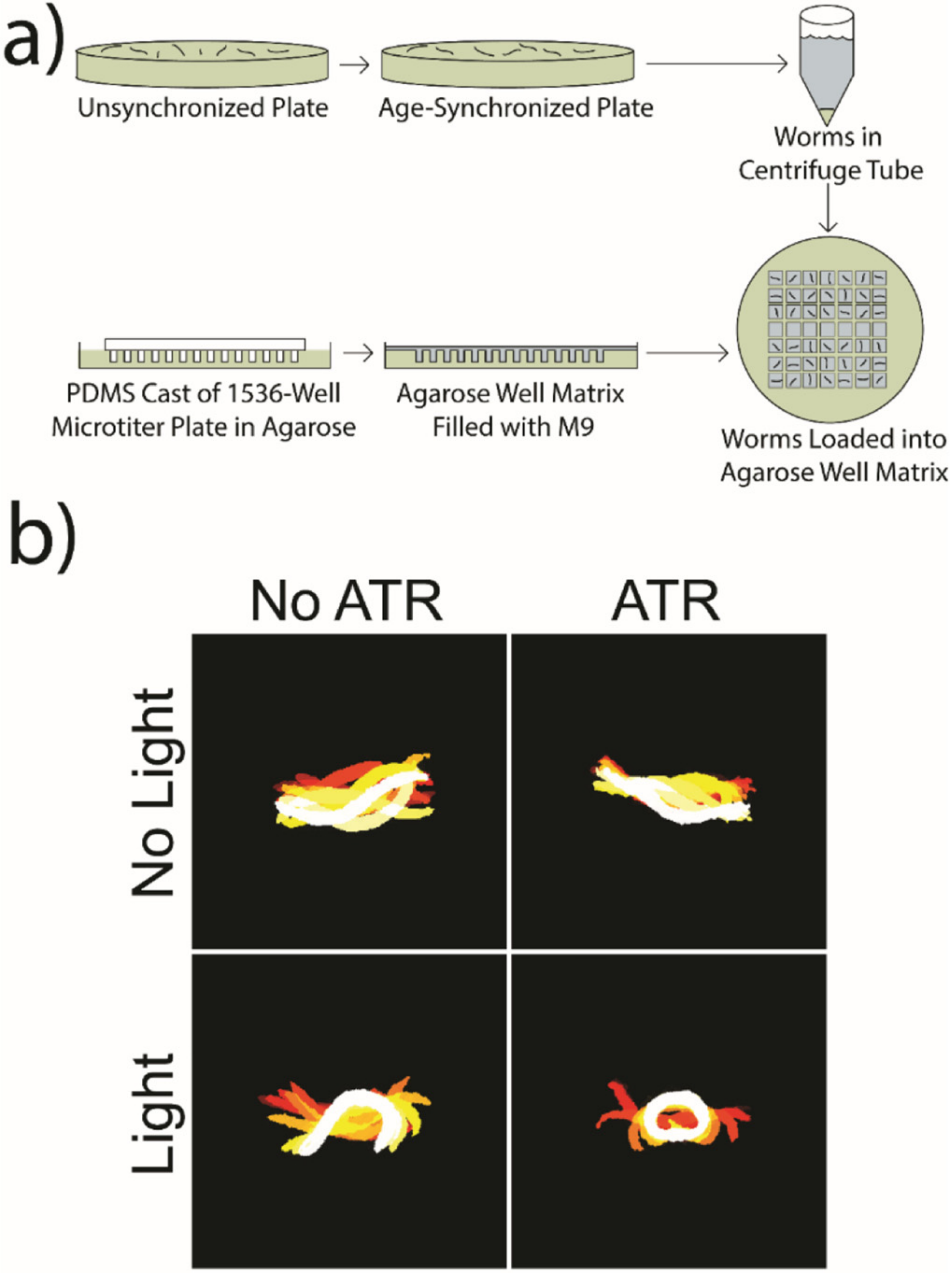
(a) An unsynchronized plate of worms is age-synchronized through treatment with hypochlorite solution. The worms are washed to a centrifuge tube and loaded manually into the prepared 3% agarose well matrix with a 10 *μ*l pipette. A 9-by-13 matrix of wells can be filled and imaged with this method. (b) Locomotion comparison assay under various conditions. Two adult ZX460 worms fed OP50 and OP50/ATR were selected from subsets of 28 worms fed OP50 and 28 worms fed OP50/ATR for purposes of comparison. Frames were taken from recorded videos at two time points: 3 s without LED lamp power and 3 s beginning with a 1 s LED lamp exposure. Each video was segmented and 11 linearly spaced frames from each 3 s video were superimposed to create the above figure. The 11 frames begin with the dark red silhouette and transition in color to the light-yellow and white silhouette on frame 11.

For specific neuronal activation regimens to be tested, the platform must induce contractions in worms under specific conditions and not stimulate the worms during the neuronal refractory period. To confirm this property of our platform, we tested the responses of animals suspended in liquid to the LED light source. We reasoned that contraction responses would be more drastic in liquid media than in typical solid media. An age-synchronized population of ZX460 animals was washed to two plates, one seeded with OP50/ATR and the other with OP50/ethanol. The worms were stored at 20 °C for 24 h and then suspended in M9 buffer in agarose wells. Worms were imaged during light on and off periods, and the video frames segmented and overlaid for locomotion visualization (Fig. 2). The baseline swimming motion observed in the OP50/ethanol fed worm and the OP50/ATR fed worm with no light exposure show that worm contraction is not due to phototaxis or response to diet. LED lamps emit negligible amounts of infrared radiation and therefore negligible thermal radiation. In addition, since the entire plate was illuminated with approximately the same intensity, no spatial thermal gradient is created, which would cause thermotactic behaviors (Jurado *et al.*, 2010). We confirmed an absence of thermal gradients by measuring temperature across plates under to various regimes of LED exposures (supplementary material, Fig. S2). The lack of locomotion change upon light exposure in the worm not fed ATR also suggests that thermotaxis is not a factor affecting locomotion in this system. However, when the OP50/ATR fed worm was exposed to ∼457 nm light for 1 s, it contracted and relaxed back to the neutral body position over a 3 s span. This was visualized by overlaying segmented worm images for each condition over a 3 s span, as seen in Fig. 2. 3 s video clips were taken from supplementary material, Video 1. Locomotion is visualized by the color progression of segmented worm silhouettes from dark red at the beginning of the clips to white at the end. Worm silhouettes were obtained through binarizing and filtering frames containing a single well/worm. The contraction is indicative of light-induced activation of excitatory cholinergic neurons, which innervate body wall muscles. These expected results confirm that worms will not undergo neuronal activation unless two controlled conditions are met. The first condition is that the moiety created by the binding of ATR to the ion channel protein is present. Without the cofactor ATR present, the channelrhodopsin will not be light-sensitive. The second condition necessary for neuronal activation is that light stimulation must be present for the light-sensitive moiety to induce a conformational change in the ion channel. These two conditions allow for specific and high-fidelity light-induced neuronal activation without a risk of accidental activation.

**FIG. 3.**
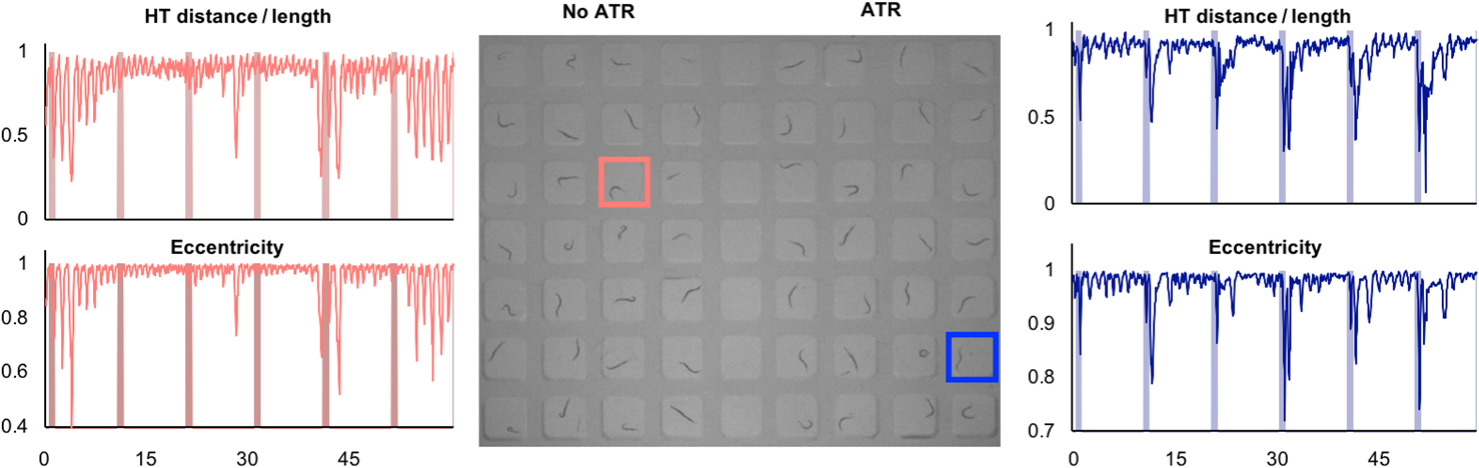
Quantification of optogenetic-driven contractions in liquid media. Left: HTOL (Head-to-tail distance over length) and eccentricity for a swimming control animal, cultured without ATR (animal under study highlighted in red in the center image). Center: A snapshot of the video for swimming animals, taken from supplementary material, Video 1. The first four columns are control animals cultured without ATR, and the last four columns are animals cultured with ATR. Right: HTOL and Eccentricity for a swimming animal cultured with ATR (animal under study highlighted in blue in the center image). Bands in graphs represent timing of light exposure. Additional traces for animals in other wells are shown in the supplementary material.

To further characterize the optogenetic-driven contraction responses in swimming animals, we performed image analysis to extract two metrics representative of animal posture: (a) eccentricity and (b) HTOL (Head-to-Tail over Length). Eccentricity was determined using MATLAB's regionprops command, returning a value of 1 for a perfectly linear worm and 0 for a perfectly circular worm. Eccentricity is calculated by fitting an ellipse to the segmented worm and dividing the distance between the ellipse's foci by length of the major axis. By examining the eccentricity over time, the animal's responses to optogenetic stimulation can be quantitatively inspected. We also analyzed contraction by HTOL, which measures the ratio of the head-to-tail Euclidean distance over the entire animal length. Representative traces for ZX460 animals cultured without (control) and with ATR are shown in Fig. 3. As can be seen from the presented traces, although the value ranges for both metrics are different, they display a similar trend representative of animal contraction. Locomotion behavior is captured for control animals, where contractions are uncorrelated with light exposure. In contrast, there is a clear correlation of contractions (dips in both metrics) following light stimulation in ATR exposed animals. Additional traces for other animals are shown in the supplementary material. Our well matrix allows for simultaneous stimulation and quantitative imaging of multiple animals in liquid media in parallel.

### C. Optogenetic-driven locomotion quantification in solid media

With confirmation of the specificity of neuronal activation, we next sought to quantitatively characterize the effects of optogenetic stimulation on worm behavior on solid media. Due to the physical result of neuronal activation in these strains, image processing serves as a useful tool to determine the extent of contractions experienced by the stimulated animals. As previously described, single worms were exposed to light and imaged simultaneously. MATLAB was used for image segmentation to obtain frames of individual organisms. Here, we also used eccentricity as a measurement of animal contraction.

**FIG. 4.**
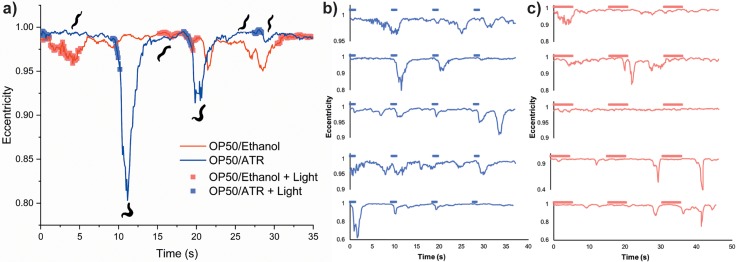
Eccentricity of young adult ZX460 worms in segmented video frames, single animal stimulation, and imaging mode. (a) Overlaid worm images are segmented images of the worm from the OP50/ATR plate (n = 1). These datasets were taken from a larger experiment (n = 25) to determine the optimal exposure settings for imaging and thus have different LED illumination patterns for the OP50/ethanol and OP50/ATR. The control OP50/ethanol data are an average of four trials and never show an eccentricity less than 0.95 as a response to LED stimulation (standard deviation of OP50/Ethanol eccentricities: σ = 0.022, n = 5). The worm from the OP50/ATR shows two eccentricity dips below the baseline response, corresponding to 2 light induced contractions. (b) Traces for 5 sample animals from an OP50/ATR plate. Blue bars on top represent LED light on. (c) Traces for 5 sample control animals from an OP50/ethanol plate. Red bars on top represent LED light on.

This approach allowed us to determine that there is minimal delay (<100 ms) between light exposure and physical response, and that subsequent exposures to light reduce the strength of the response. When imaged, the worms containing ChR2(H134R) showed a strong response on the first LED lamp stimulus, but decreasing contraction strengths on each subsequent stimulus, as shown by the increasing eccentricity values. Worms from OP50/ethanol plates were also imaged to determine the quantified response to light exposure, and it was seen that the eccentricity of each worm did not decrease below ∼0.95 for normal crawling. The average eccentricity of the OP50/ethanol worms was 0.978 with standard deviation equal to σ = 0.022 (n = 5). This low standard deviation allows for designation of troughs dipping below 0.956 as successful contractions, as data lower than this are more than one standard deviation below the mean. For a pair of OP50/ATR and OP50/ethanol worms, the OP50/ATR worm showed two responses, leading to eccentricities below 0.956, indicating two successful neuronal activations (Fig. 4). Traces for five sample animals (ZX460) and five control animals are shown in Figs. 4(b) and 4(c). While the values can vary among experiments, a change in eccentricity is evident in most scenarios after light exposure for animals cultured on ATR plates. In contrast, control animals show dips in eccentricity, but these are uncorrelated with the timing of the light exposure, indicating natural behaviors (such as omega bends). In this mode, the platform images a single worm during normal locomotion on plate, and thus the resultant studies have a low n-number. In this mode of operation, it is possible for animals to escape the field of view, in which case the stage is manually adjusted. While this limitation of our system impacts throughput and could be overcome by a feedback system to control the stage automatedly, we aimed to develop a simple, easy to implement platform.

### D. Synaptic plasticity

**FIG. 5.**
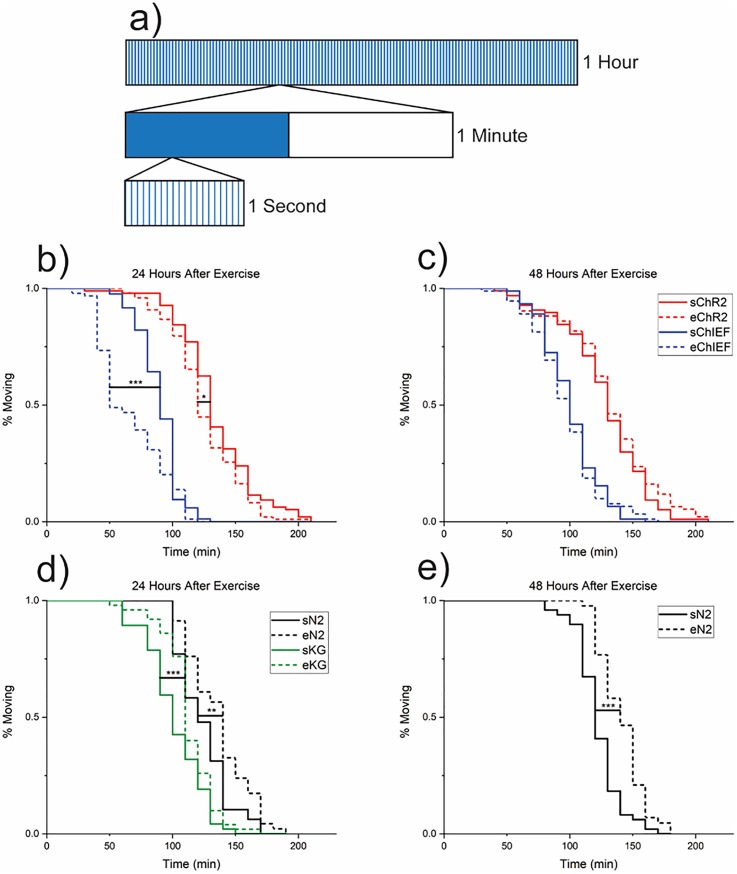
Synaptic plasticity assay setup and results. (a) Visualization of the stimulation regime. Each of the 20 blue sections in the 1 s bar is a 10 ms light exposure. (b) Aldicarb paralysis assays for exercised and sedentary populations of ChR2(H134)-containing strain ZX460 and ChIEF-containing strain oxSi91. Assay performed 24 h after exercise, animals are the same age. Two trials consisting of exercised and nonexercised populations for both ZX460 and EG5793 were taken using 2 biological replicates for each group (n = 20–25 per replicate). % moving data for each replicate and trial were averaged. (^***^ = p ≤ 0.001, ^*^ = p < 0.05, Logrank test). (c) Same as “b” but 48 h after exercise. Difference between curves for each strain is nonsignificant. (d) Aldicarb paralysis assays for wildtype N2 and lite-1 mutant KG1180 exercised and sedentary populations. One trial consisting of exercised and nonexercised populations for both N2 and KG1180 was taken using 2 biological replicates for each group (n = 20–25 per replicate). % moving data for each replicate were averaged. (^***^ = p ≤ 0.001, ^**^ = p < 0.01, Logrank test). (e) Same as “d” but 48 h after exercise, only N2 replicates are represented. Solid lines represent sedentary worm populations, and dotted lines represent exercised worm populations. In the legend, the s- and e-prefixes denote sedentary and exercised. Strains are represented by line color.

We next sought to apply our platform to assess synaptic plasticity in *C. elegans* cholinergic motoneurons. Specifically, we applied the platform to determine whether regimens of optogenetic neuronal stimulation induce changes in synaptic strength. To do this, we exercised animal populations of the ChR2(H134)-containing ZX460 and ChIEF-containing EG5793 strains. These were cultured as previously described and divided into sedentary and exercised populations. The exercised worms underwent an hour-long stimulation regimen consisting of 10 ms pulses of ∼457 nm light at 20 Hz for the first 30 s of every minute, as shown in Fig. 5(a). To determine whether this neuronal stimulation regimen induces changes in synaptic function, we used an aldicarb paralysis assay. Aldicarb acts as a cholinesterase inhibitor, which prevents acetylcholine in the synapse from being broken down and recycled. The accumulation of acetylcholine in the synaptic cleft causes inundation of the postsynaptic receptors, ultimately leading to animal paralysis (Mahoney *et al.*, 2006). An increase in synaptic strength leads to an increase in acetylcholine emission, which should cause an increased rate of paralysis (Oh and Kim, 2017). While increased acetylcholine emission is the primary suspect for increased paralysis rates, decreased GABAergic transmission caused by high cholinergic cell activity cannot be ruled out. Locomotion in all strains was qualitatively unaffected by exercise, suggesting reduced GABAergic neuron activity is unlikely. We compared the paralysis rates of exercised and sedentary populations 1 and 2 days after stimulation to assess motoneuron synaptic plasticity. The exercised populations were picked to the aldicarb plates 24 and 48 h after stimulation (48 and 72 h after picking to OP50/ATR plates) and observed every 10 min until all animals were paralyzed. The sedentary populations were picked to the aldicarb plates 24 and 48 h after the corresponding experimental populations were exercised.

At 24 h after exercise, the effects of the stimulation regimens were apparent. The exercised population of the strain containing the ChIEF channelrhodopsin showed a steep decrease in the percentage of nonparalyzed worms, going from 95% moving to 80% paralyzed from the 30- to 90-min marks. The sedentary ChIEF population had a much slower locomotion falloff, going from 95% moving to 80% paralyzed from the 50- to 100-min marks. The gap between the two curves indicates a higher strength of synaptic transmission in the exercised population (p = 2 × 10^–5^, Logrank). Both the exercised and sedentary ChR2(H134) populations showed a much slower rate of paralysis than either ChIEF population, with the sedentary ChR2(H134) population being comparable to the sedentary N2 population. The exercised ChR2(H134) population showed a significantly quicker locomotion decline than sedentary population (p < 0.05, Logrank), as seen in Figs. 5(b) and 5(d). The observed difference between the two strains is most likely due to asymmetric growth rates between strains. We observed that EG5793 grows at a slower rate than ZX460, which could be attributed to either oxSi91 reducing animal fitness or potentially EG5793 being prone to mutations. While the two strains were age synchronized at the time of exercise, the growth rate differential leads to an asynchronous baseline response at the time of the assay. In addition, the differences between exercised strains could be explained by a combination of ChR2(H134) channelrhodopsin's slower kinetics and the short length and high frequency of light pulses in the stimulation regimen used. ChIEF shows >95% successful light-induced action potentials with 25 Hz light exposure, but ChR2(H134) only shows slightly greater than a 50% success rate in action-potential induction (Lin, 2010; Lin *et al.*, 2009).The results of the strains containing channelrhodopsins are even more stark when compared to those of N2 and KG1180. Both N2 and KG1180 show a significantly faster (p = 0.002, p = 1 × 10^–5^, Logrank) paralysis in the sedentary populations compared to the exercised ones as seen in Fig. 5(d). This indicates some mechanism, whether sensitivity to aldicarb or general locomotion change, is triggered by the stimulation regimen, which decreases paralysis rates in exercised strains. The exercised strains containing channelrhodopsins are able to overcome this effect, as the exercised populations are paralyzed significantly faster than the sedentary ones.

The results 48 h after stimulation show a more muted difference between strains and populations. The sedentary populations from each strain were paralyzed at nearly the same rate, with the ChIEF population being paralyzed slightly slower. The exercised ChIEF strain shows no significant difference from the sedentary strain. The progression from a highly significant to minimal difference in ChIEF populations from 24 to 48 h suggests that the exercise-induced strengthening of synapses is not permanent and transiently declines with time. These results suggest that cholinergic motoneurons exhibit synaptic plasticity, where strengthening by neuronal stimulation is possible but temporary. The exercised ChR2(H134) strain also showed a slower locomotion falloff as compared to the 24-h mark, as seen in Fig. 5(c). While this result is nonsignificant as determined by the Logrank test, the response of this strain was significant at 24 h after exercise, indicating behavioral divergence over time. When comparing behavior at 24 and 48 h after exercise, it was seen that the exercised populations curves shift rightwards over time, and the sedentary populations show minimal change. The exercised population of N2 showed slightly slower paralysis at 48 h compared the 24-h mark, but the sedentary population showed a negligible change (p = 0.001). Given that the same trend holds at the 48-h mark for N2, but the effects of stimulation are diminished in the strains with channelrhodopsins, we conclude that the effects of stimulation in optogenetically active strains are transient and diminish over time.

## III. DISCUSSION

In this work, we present an integrated platform that enables optogenetic stimulation and quantitative locomotion analysis of *C. elegans.* The platform we created is inexpensive and simple to setup, allowing for easy entry into the field of optogenetics. By replacing mercury lamp/laser systems with a high-power LED and pattern generators with a MATLAB script and an Arduino board, the cost typically associated with neuronal stimulation and synaptic plasticity studies is cut significantly, while not limiting system customizability. The MATLAB controlled Arduino platform can drive optogenetic neuronal exercise, image, and analyze animal contraction. This system enables high-throughput behavioral studies pertaining to synaptic transmission, strength, and plasticity. This robust platform offers an expansive array of neuronal stimulation regimens for a fraction of the cost of most current systems. Despite the platform's economical nature, it returns reliable quantitative data, which can be used to probe the nature of synaptic plasticity.

Using this platform to quantify the effects of optogenetic-driven locomotion, we detected contractions of decreasing magnitude on successive stimuli. We propose this is due to decreasing amounts of extracellular Ca^2+^ required for firing action potentials and/or decreasing amounts of intracellular acetylcholine (Gao and Zhen, 2011). After neuronal exercise, the strain expressing the ChIEF channelrhodopsin variant showed a higher level of synaptic transmission as compared to the nonexercised worms and the strain expressing the ChR2(H134R) variant with slower kinetics. These effects were reduced to a nonsignificant level 48 h after the neuronal stimulation, suggesting that synaptic strengthening of motoneurons is a transient effect. The difference in the effect of neuronal stimulation on the optogenetically active strains compared to N2 and KG1180 indicates blue light exposure alone decreases synaptic strength as determined by the aldicarb assay. The presence of channelrhodopsin proteins causes stimulation regimens to overcome and surpass the effect of decreased synaptic strength resulting from blue light exposure, thus confirming this approach is effective for analysis of synaptic plasticity. The platform developed will be useful to test a wider range of stimulation regimens to assess the limits of synaptic plasticity and its interplay with aging. For instance, it would be interesting to determine whether periodic stimulation is able to maintain synaptic strengthening for longer or if the synapses can become stronger with daily stimulation regimens.

Our platform presents utility in a variety of aspects of optogenetic studies for *C. elegans* on solid and liquid media. On-plate optogenetic-driven locomotion can be inexpensively explored and quantified for a low number of animals through synchronized optogenetic stimulation and image recording. This aspect can be used for determining potential stimulation regimens and investigating behavioral changes due to optogenetic stimulation. Our platform also enables highly programmable optogenetic-driven neuronal stimulation for entire populations of animals. This enables performing studies dealing with synaptic plasticity for large populations.

This work focused on stimulation of cholinergic neurons as the induced locomotory effects are easily discernable. An important question we aim to answer in future work is whether these effects differ in liquid vs solid media. Moreover, any neuronal promoter for other classes of neurons could be used and subsequently exercised to investigate the downstream behavioral and synaptic effects. For instance, controlled neuronal stimulation regimens could be tested in mechanosensory neurons, where escape response (rather than contraction) is assessed. In addition, in future work, we aim to determine whether exposure to neuronal stimulation regimens at various life stages has an effect on synaptic connectivity and function in aged individuals. Neuronal stimulation could be induced or examined at various time points throughout the development of *C. elegans*, which could be especially useful in age-related neurodegenerative diseases such as Alzheimer's disease and Parkinson's disease. Notably, this platform enables exposing large populations to optogenetic-drive neuronal exercise, thus allowing coupling this system with downstream analysis, such as analysis of synaptic patterning and morphology through fluorescent markers, in a high-throughput fashion.

## IV. METHODS

### A. Strains and culturing

Worms were age synchronized by harvesting eggs from gravid hermaphrodites using a 0.3 M hypochlorite and 0.5 M NaOH solution and then washed using M9 buffer before plating. Worms were plated on nematode growth medium (NGM) plates seeded with OP50 *Escherichia coli* and cultured at 20 °C (Brenner, 1974). For all experiments, 60 mm plates were used. 24 h before imaging, worms were picked to plates seeded with a mixture of all-trans retinal (ATR) and OP50. The OP50/ATR mixture consisted of a 100-fold dilution of 1 mM ATR in ethanol and OP50 cultured overnight in Luria-Bertani broth. The seeded plates were incubated in the dark at room temperature for 48 h before use. Worms used in control groups were picked to plates with OP50 solutions containing 1% ethanol, which were seeded and incubated in the same manner. Strain ZX460, developed by the Gottschalk Laboratory was obtained from the Caenorhabditis Genetics Center (CGC). This strain contains transgene *zxIs6 [Punc-17::ChR2(H134R)::YFP + lin-15(+)]* and expresses the channelrhodopsin variant ChR2(H134R) in cholinergic neurons under the *unc-17* promoter, which encodes a synaptic vesicle acetylcholine transporter (Liewald *et al.*, 2008). Strain EG5793, provided by the Jorgensen Laboratory, *oxSi91[Punc-17::ChIEF::mCherry::unc-54UTR; unc-119*(+)] *II* was also used due to the more consistent action potential induction of the chimeric channelrhodopsin variant ChIEF (Hoerndli *et al.*, 2015; Lin, 2010; Lin *et al.*, 2009; Watanabe *et al.*, 2013). Bristol wild type strain N2 was used as a control in the aldicarb paralysis assay. Since exposure to blue light can induce phototaxis via ultraviolet light receptor protein LITE-1, strain KG1180 developed by the Miller Laboratory with genotype *lite-1(ce314)* was used in the aldicarb paralysis assay to assess the effects of increased locomotion due to phototaxis (Edwards *et al.*, 2008).

### B. Microscopy and system setup

A high-resolution microscopy system was used to image worms, consisting of a Leica M135 FC microscope coupled with a Lumenera Infinity3–6URC CCD camera with an overall magnification of 4×. Using MATLAB, worms were imaged on plate under brightfield illumination through manual location and occasional stage adjustment to keep freely moving animals in frame. MATLAB 2018b was used, and our MATLAB script used the Image Processing Toolbox, Image Acquisition Toolbox, MATLAB Support Package for Arduino Hardware, and Yair Altman's “export_fig.” Worms were exercised using a MATLAB controlled Arduino Uno along with a triemitter high-power LUXdrive Indus Star Royal Blue LED lamp (A008-EROY0–16), with a dominant emitted wavelength between 450 and 465 nm. An Arduino Uno was used in conjunction with a SPST relay (Digi-Key CLA274-ND) to control the LED lamp. A DC power supply was used to provide 12 V to the SPST relay, which output to a 350 mA constant-current regulated LED driver (BuckToot 7027-D-350) to provide a consistent 1.65 W LED lamp power output. For single animal stimulation and analysis, a 1 s LED lamp exposure was used followed by 8 s of lamp off-time for a total of 60 s. During imaging, an mCherry emission filter with a maximum transmittance at 610 nm was used between the objective to prevent LED blue light from saturating the camera and to maintain a constant illuminance. All code is available at https://github.com/asanmiguel/CrawfordLED_Optogenetics.

### C. Image analysis

24 h before imaging/stimulation, worms are transferred to plates with OP50/ATR. Prior to imaging, worms were picked to fresh NGM plates seeded with OP50 to reduce crowding. Worms were imaged as described in Sec. IV B, and captured videos were then analyzed through MATLAB. Thresholding values for image segmentation were found by gradually decrementing the global threshold level determined by Otsu's method (graythresh function in MATLAB), until the moving worm's silhouette is well defined (Otsu, 1979). The minimum allowed object area was then incremented until all nonworm identified objects are removed. The objects are then skeletonized and analyzed to determine the eccentricity of the worm in each frame. Eccentricity is calculated using the “regionprops” function in MATLAB, which finds the ratio of the distance between the foci and major axis length of the ellipse that has the same second moments as the worm skeleton. The eccentricity was plotted over time and compared with the status of the light to determine response to light stimuli. All code and a sample video are available at https://github.com/asanmiguel/CrawfordLED_Optogenetics.

### D. Liquid media locomotion profile

To test optogenetic responses of animals in liquid media, worms were isolated in individual 3% agarose wells filled with M9 buffer. Wells are cast from 1536 well plates by first casting a polydimethylsiloxane (PDMS) mold on a 1536 microtiter plate. The PDMS mold is then used to cast the agarose wells [Fig. 2(a)]. 28 OP50/ATR-fed and 28 OP50-fed adult worms were loaded into individual wells of the 3% agarose well matrix. A 60 s video of swimming animals was recorded using periodic 1 s light pulses to observe response to light stimuli. Representative video frames for one animal from each experimental group were selected and segmented for a 2 s duration during a light-off phase and a 2 s duration beginning when the LED lamp turns on. An array of 11 frames was taken from both sets of 2 s videos and overlaid to visualize worm locomotion [Fig. 2(b) and supplementary material, Video 1]. This was captured with LUMENERA INFINITY CAPTURE version 6.5.4. This software was required to use a camera with a wider field-of-view, which was incompatible with MATLAB.

Quantification of animals in liquid media was performed by the following steps in MATLAB: each frame of the video was first divided into 63 regions, using known coordinates and width and height of each well (150 pixels × 150 pixels). Each well image was then processed using background subtraction, a median filter, image inversion, and thresholded using a value of the 98.75 percentile of the pixel values. The detected animal was then skeletonized, and endpoints were detected using “bwmorph.” Eccentricity and the length of the animal were calculated using regionprops.

### E. Stimulation and aldicarb paralysis assay

A 100 mM aldicarb solution was added to NGM solution prior to pouring plates to create NGM plates containing 1 mM aldicarb. The aldicarb assay as described by Oh and Kim is capable of detecting increased or decreased levels of synaptic transmission, which is related to speed of paralysis (Oh and Kim, 2017). The aldicarb-containing NGM plates were then seeded with OP50 and allowed to incubate for 2 days at room temperature. Worm populations were age-synchronized and cultured separately on NGM plates seeded with OP50 as previously described, before being picked to plates seeded with OP50/ATR at the L4 stage. Worms were aged for 24 h at 20 °C before exercise. The worm stimulation regimen consisted of 1 h of 10 ms light pulses at 20 Hz for the first 30 s of every minute (Hoerndli *et al.*, 2015). 24 h after exercise, 20–25 worms were picked to the aldicarb-containing NGM plates and observed every 10 min. Additional worms from the same populations were then picked to aldicarb plates 48 h after exercise. Worms were prodded to confirm paralysis, and the assay continued until no worms responded to touch. Worms suspected of being paralyzed were prodded with a platinum wire and were considered paralyzed after 5 s of further observation. Two trials were performed with two biological replicates of strains ZX460 and EG5793, and one trial was performed with two biological replicates of N2 and KG1180. Statistical comparisons between survival curves were performed using Therneau's “survival” package in R (R version 3.5.1).

No ethics approval is required for this work.

## SUPPLEMENTARY MATERIAL

See the supplementary material for additional information and data, including (1) a picture of our system setup (Fig. S1), (2) measurement of temperature on NGM plates with and without a lid after different light exposure regimens (Fig. S2), (3) quantitative analysis of optogenetic-driven contraction and locomotion in swimming animals (Figs. S3 and S3), and (4) a video of single animals swimming in the agarose well matrix in the absence and presence of ATR (supplementary material, Video). A description of the temperature measurement and a description of the video are also included.
